# Biomarkers of Drug Resistance in Temporal Lobe Epilepsy in Adults

**DOI:** 10.3390/metabo13010083

**Published:** 2023-01-04

**Authors:** Yulia S. Panina, Elena E. Timechko, Anna A. Usoltseva, Kristina D. Yakovleva, Elena A. Kantimirova, Diana V. Dmitrenko

**Affiliations:** Department of Medical Genetics and Clinical Neurophysiology, Institute of Postgraduate Education, V.F. Voino-Yasenetsky Krasnoyarsk State Medical University, 660022 Krasnoyarsk, Russia

**Keywords:** temporal lobe epilepsy, biomarkers, drug resistance, neuroinflammation, neurodegeneration

## Abstract

Temporal lobe epilepsy (TLE) is the most common type of focal epilepsy in adults. Experimental and clinical data indicate that neuroinflammation and neurodegeneration accompanying epileptogenesis make a significant contribution to the chronicity of epilepsy and the development of drug resistance in TLE cases. Changes in plasma and serum concentrations of proteins associated with neuroinflammation and neurodegeneration can be predictive biomarkers of the course of the disease. This study used an enzyme-linked immunosorbent assay of the following plasma proteins: brain-derived neurotrophic factor (*BDNF*), tumor necrosis factor alpha (*TNFa*), and high-mobility group protein B1 (*HMGB1*) in patients with mesial TLE to search for biomarkers of the disease. The objective of the study was to examine biomarkers of the neuroinflammation and neurodegeneration of plasma: *BDNF*, *TNFa*, and *HMGB1*. The aim of the study was to identify changes in the concentration of circulating pro-inflammatory and neurotrophic factors that are prognostically significant for the development of drug resistance and the course of TLE. A decrease in the concentration of *BDNF*, *TNFa*, and *HMGB1* was registered in the group of patients with TLE compared with the control group. A significant decrease in the concentration of HMGB1 in patients with drug-resistant TLE was observed. Aberrations in plasma concentrations of *BDNF*, *TNFa*, and *HMGB1* in patients with TLE compared with the controls have been confirmed by earlier studies. A decrease in the expression of the three biomarkers may be the result of neurodegenerative processes caused by the long course of the disease. The results of the study may indicate the acceptability of using *HMGB1* and *TNFa* as prognostic biological markers to indicate the severity of the disease course and the risk of developing drug resistance.

## 1. Introduction

Temporal lobe epilepsy (TLE) is characterized by recurrent seizures with an onset involving the amygdalohippocampal complex and parahippocampal place area (PHA) [1]; it is the most common type of focal epilepsy in adults [2].

Approximately 30–50% of patients with TLE develop drug resistance [3]. According to the International League Against Epilepsy (ILAE) [4], drug-resistant epilepsy is defined “as failure of adequate trials of two tolerated and appropriately chosen and used antiepileptic drugs (AED) schedules (whether as monotherapies or in combination) to achieve sustained seizure freedom”. One of the major causes of drug resistance in temporal lobe epilepsy is the presence of hippocampal sclerosis (HS) [5]. Hippocampal sclerosis is caused by: a decrease in the volume of the hippocampus; dystrophic changes in neurons and a decrease in their number (mainly in the CA1 layer), relative to an increase in the number of glial cells; and demyelination of fibers [6]. Hippocampal sclerosis is characterized by the presence of the following signs according to MRI data: an increased signal from the structure in T2 and T2 FLAIR modes; and a reduction in the size of the structure in the T1 and T1 “inversion-recovery” modes by more than 30% [7].

Factors that often lead to the development of TLE (trauma, tumor, infection, etc.) are accompanied by massive neuroinflammation and neuronal loss [8,9]. Inflammation, as already known, significantly contributes to the pathogenesis of epilepsy [10]. Post-seizure inflammation can be a physiological response to stress and brain tissue damage, helping in its reparation and in balancing the enhanced metabolic demand during increased synchronic neuronal activity. Nonetheless, if the neuroinflammatory response is protracted or too intense, it can become maladaptive, leading to cellular dysfunction that is seen in epilepsy and other neurological diseases [11]. The expression of inflammatory mediators, which are induced by seizure activity in astrocytes and microglia, can cause a cascade of subsequent inflammatory events [12,13,14]. This leads to a change in the excitability of neuronal networks, which increases the likelihood of seizures [11] and further neurodegenerative processes [15].

Experimental and clinical data demonstrate that neuroinflammation and neurodegeneration involved in epileptogenesis make a significant contribution to the development of drug resistance and the chronicity of TLE [16,17]. The mechanism of development of neuroinflammation and neurodegeneration in TLE has been studied in experimental models as well as in clinical samples of biological fluids and tissues of patients [17,18,19]. In TLE cases with hippocampal sclerosis (HS), there is a progressive loss of neurons and gliosis in the hippocampus and amygdala, which may be caused by uncontrolled processes of neuroinflammation and neurodegeneration accompanied by disruptions in the blood–brain barrier and damage to brain cells [20].

Neuroinflammation can break the permeability of the blood–brain barrier, so the peripheral inflammatory status should reflect some characteristics of the central inflammatory status. Peripheral biomarkers of neuroinflammation have been proposed, [21] although a clear comparison between central and systemic inflammation is still lacking.

A problem in modern neurology is the search for a significant association between the biomarkers of neuroinflammation and the neurodegeneration that occurs during the course of the pathological process at different stages of TLE, which would permit the prediction of drug resistance and selection of further treatment tactics [22,23].

Protein biomarkers for various neurological diseases [24,25] have been previously discussed, as have other molecular biomarkers for temporal lobe epilepsy [26] in earlier studies.

The objective of the study was to identify prognostic biomarkers for the course of TLE and the risk of developing drug resistance.

## 2. Materials and Methods

The study was carried out as part of a comprehensive study on the topic “Management of orphan diseases” under registration number AAAA-A19-119031990004-3 at the Department of Medical Genetics and Clinical Neurophysiology of the Institute of Postgraduate Education of Prof. V.F. Voino-Yasenetsky Krasnoyarsk State Medical University, Russia.

The study was conducted according to the guidelines of the Declaration of Helsinki and was approved by the Local Ethics Committee of Prof. V.F. Voino-Yasenetsky Krasnoyarsk State Medical University (extract from protocol No. 85/2018, dated 27 September 2018).

### 2.1. Patients

We consecutively enrolled TLE patients with hippocampal sclerosis (HS) based on the following criteria: (1) diagnosis of epilepsy, (2) history of focal seizures consistent with TLE semiology, and (3) typical brain MRI features of HS, including increased signal intensity on fluid-attenuated inversion recovery (FLAIR) imaging with atrophy in the hippocampus.

TLE and drug-resistant TLE were diagnosed by a professional specializing in the neurology of epileptic disorders and epilepsy neuroimaging.

Patients were included in the study upon signing an informed consent form.

Experimental group inclusion criteria:

Patients with a confirmed diagnosis of mesial TLE;

Age from 18 to 60 years old;

Residents of the Siberian Federal District;

Patients who voluntarily signed the informed consent form.

Experimental group exclusion criteria:

Patients with confirmed focal genetic and other forms of epilepsy;

Lack of voluntarily signed informed consent;

An increase in body temperature during the analysis, as well as the presence of signs of an infectious disease suffered less than a month before the analysis;

Exacerbation of a chronic disease;

Concomitant somatic diseases in the decompensation stage.

Control group: 203 volunteers, matched in age and sex with the patients of the main group.

Control group inclusion criteria:

People aged from 18 to 60 years old;

Patients who voluntarily signed the informed consent form.

Control group exclusion criteria:

Presence of a neurological or psychiatric disorder;

Subclinical epileptiform changes on the EEG;

An increase in the body temperature during the analysis, as well as the presence of signs of an infectious disease suffered less than a month before the analysis;

Exacerbation of a chronic disease;

Lack of voluntarily signed informed consent;

Alcohol and/or drug addiction;

Concomitant somatic diseases in the decompensation stage.

The median age of patients was 35.0 [29; 46] years. Sex of patients: 69 males (41.6%) and 97 females (58.4%).

The age of the onset of the disease was 19.0 [13; 30.5] years. The median duration of the disease in patients was 11.0 [9.0; 20.0] years. The severity of epileptic seizures according to the National Hospital Seizure Severity Scale (NHS-3) ranged from 1 to 23 points, where the median was 13.0 [11.0; 16.0] points. Hippocampal sclerosis was confirmed in 62 patients by brain MRI imaging.

The main characteristics of the patients are compiled in Table 1.

### 2.2. Methods

Clinical interrogation involved the study of the neurological status, assessment of the frequency and severity of epileptic seizures (including using the NHS-3), analysis of ongoing antiepileptic therapy, and response to AEDs. The study of neurological status was conducted according to the NHS-3, EEG video monitoring, and MRI imaging of the brain.

The determination of the concentration of *BDNF* and *TNF-α* in blood plasma was carried out using ElisaKit kits: SEA011Hu, SEA133Hu, SEC183Hu, SEA563Hu (Cloud-Clone Corp., Katy, TX, USA) and a model 3300 StatFax biochemical analyzer.

The immunobiochemical study of *HMGB1* proteins in blood plasma was carried out using an enzyme-linked immunosorbent assay using the Luminex fluorescent technique with multiplex magnetic beads (Milliplex MAP Human Cytokine/Chemokine Magnetic Bead Panel IV).

### 2.3. Data Analysis

The median and 25–75% (Me [LQ; UQ]) were used to describe the amount of data with abnormal accumulation. The study used percentages and 95% confidence intervals (95% CI) to describe the statistics. A non-parametric analysis of variance (the Kruskal–Wallis test) followed by a posteriori pairwise comparison of the groups with each other were carried out to compare several groups on a quantitative basis. The Mann–Whitney test was carried out to compare the two groups.

The odds ratio (OR, 95% CI) or hazard ratio (HR, 95% CI) were used to assess the risk factors associated with the development of TLE. Spearman’s correlation coefficient (r) was used to assess the relationship between quantitative traits with non-normal distribution. Intergroup differences were recognized as statistically significant at *p* < 0.05.

An ROC analysis was used with the determination of the area under the curve (AUC) and results being statistically significant at *p* < 0.05 to assess the quality of the classification. A logistic regression was used as a forecast to assess the influence of a combination of factors.

## 3. Results

In the group of patients with TLE, a lower concentration of *BDNF, TNFa*, and *HMGB1* was detected compared with the control group (25.87 [20.81; 32.17] ng/mL vs. 74.85 [45.11; 128.85] ng/mL, *p* < 0.001, 12.30 [10.27, 20.95] pg/mL vs. 73.40 [56.42, 92.88] pg/mL, *p* < 0.001 and 135.765 [114.17, 159.53] pg/mL vs. 161.73 [136.34; 179.01] pg/mL, *p* = 0.034, respectively) (Table 2 and Table 3).

In all patients with TLE, there was a decrease in the concentration of *BDNF* and *TNFa* (Table 2). The decrease did not depend on the response to antiepileptic drugs, mono- or polytherapy of AEDs, or the presence of hippocampal sclerosis (*p* > 0.05).

**Figure 1:**
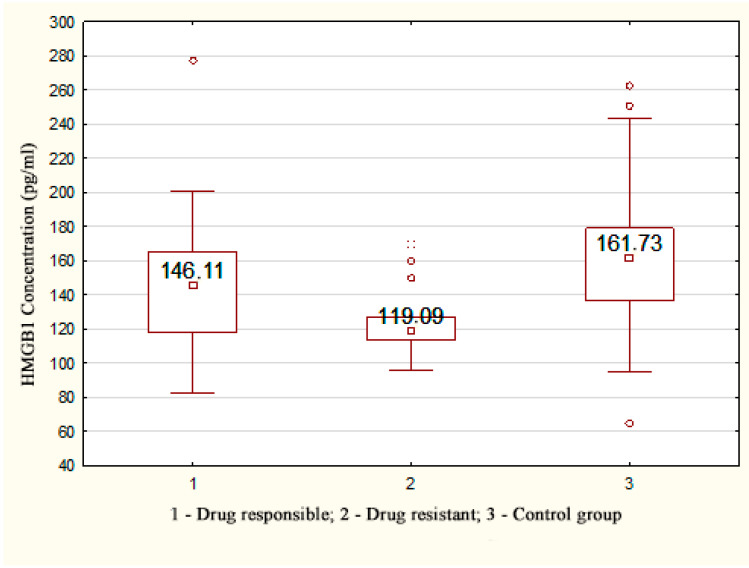
*HMGB1* concentration depending on the response to therapy in patients with TLE.

A statistically significant decrease in the concentration of *HMGB1* was registered in patients with drug-resistant TLE (119.09 [113.55; 126.93] pg/mL) compared with the control group (161.73 [136.34; 179.01] pg/mL), *p* < 0.001; a statistically significant decrease was also observed in patients without drug resistance (146.11 [117.87; 165.56] pg/mL), *p* = 0.049 (Figure 1).

There was no relationship between the *HMGB1* level and the clinical characteristics of epilepsy (the course of TLE, type of structural disorders, nature of EEG changes, type of AED therapy, and duration of TLE) (*p* > 0.05).

A statistically significant decrease in the concentration of *TNFa* was registered in patients with a TLE duration of more than 10 years (10.58 [8.70; 16.30] pg/mL) and a disease duration of less than 10 years (14.30 [11.22; 26.85] pg/mL) compared with the control group (*p* < 0.001).

**Figure 2:**
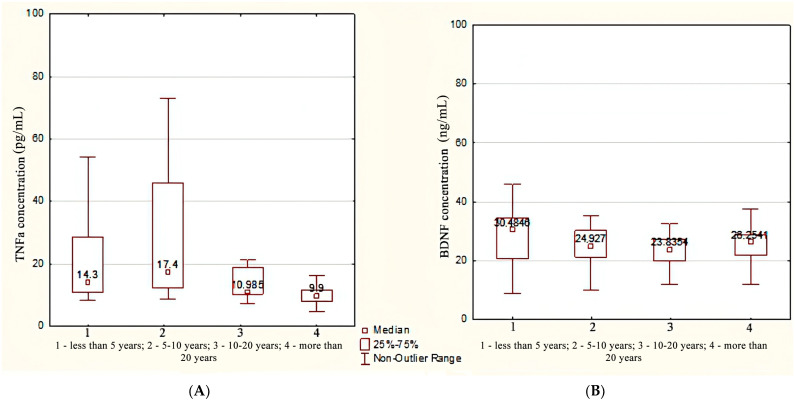
The concentration of *TNFa* (**A**) and *BDNF* (**B**) depending on the duration of TLE.

It was found that the concentration of *TNFa* in patients with a TLE duration of more than 20 years (9.90 [7.98; 11.60] pg/mL) was statistically significantly lower compared with patients with an epileptic seizure experience of up to 5 years (14.30 [11.00; 28.50] pg/mL, *p* = 0.009) and 5–10 years (17.40 [12.30; 45.90] pg/mL, *p* = 0.006) (Figure 2A).

In patients with a TLE duration of less than 5 years, the *BDNF* concentration was higher compared with patients with a duration of epileptic seizures in the range of 10–20 years (30.4 [20.8; 30.4] ng/mL vs. 23.8 [19.8; 27.3] ng/mL), *p* = 0.025 by median test. However, according to the Kruskal–Wallis test followed by a pairwise comparison using the Mann–Whitney test, there were no statistically significant differences, *p* = 0.068 (Figure 2B).

To assess the contribution of changes in the concentration of *BDNF* and *TNFa* to the pathological process, an ROC analysis was performed.

**Figure 3:**
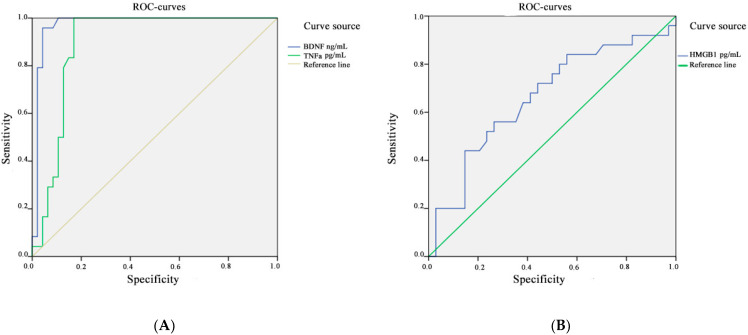
ROC analysis for the concentration of *BDNF* and *TNFa* (**A**) and *HMGB1* (**B**) in the blood plasma of patients with TLE and in the control group.

The area under the ROC curve for *BDNF* and *TNFa* was 0.975, CI 95% [81.12; 97.82%] and 0.895, CI 95% [63.56%; 98.54%], respectively (*p* < 0.001). The threshold value for *BDNF* at the cut-off point was 37.5 ng/mL, and the value for *TNFa* was 33.5 pg/mL; values equal to or less than this indicator were recorded in patients with a long experience of TLE. The sensitivity for *BDNF* concentration was 92.16%, and the specificity was 88.24%. The sensitivity for *TNFa* concentration was 93.94%, and the specificity was 88.89% (Figure 3A).

The area under the ROC curve, which corresponds to the relationship between the development of drug-resistant TLE and the concentration of *HMGB1*, was 0.664, CI 95% [45.12%; 79.60%]; the threshold value at the cut-off point was 146.5 pg/mL. Values equal to or below this indicator predict a high risk of developing a drug-resistant course of epilepsy. The resulting models were statistically significant (*p* < 0.001). The sensitivity for *HMGB1* concentration was 63.64%, and the specificity was 60.00% (Figure 3B). The results of the ROC analysis are compiled in Table 4.

There is no correlation between the severity of epileptic seizures on the NHS-3 scale and the levels of *BDNF*, *TNFa*, and *HMGB1* (Spearman’s correlation coefficient r = −0.14, −0.14, and 0.15, respectively, *p* > 0.05).

## 4. Discussion

Aberrations in the plasma markers of neuroinflammation and neurodegeneration observed in our study have also been found in a number of previous studies, which are described in the literature. Our research revealed a lower concentration of *BDNF*, *TNFa*, and *HMGB1* proteins in the group of patients with TLE compared with the control group. There were no statistically significant differences in other markers associated with epileptogenesis in the group of patients with TLE compared with the control group.

A number of studies have shown a correlation between the severity of epileptic seizures and increased levels of cytokines in the blood plasma (*HMGB-1*, *TLR4*, *IL-1β*, *IL-1R1*, and *TNF-α*) of patients with drug-resistant epilepsy [17,27]; increased levels of cytokines have also been observed in cerebrospinal fluid (*IL-1β*) [16,28]. The impairment of the profile of pro-inflammatory cytokines in plasma (*IL-1β*, *IL-6*, *IL-8*, *TNF-α*) also correlates with the frequency of seizures [29]. However, the level of cytokines has not been studied early when monitoring the effectiveness of treatment in TLE cases [29,30].

It has also been reported that pro-inflammatory cytokines are upregulated in patients with TLE compared with patients with extratemporal epilepsy [31,32,33].

We found a decrease in the level of *HMGB1* concentration in patients with drug-resistant TLE compared with the control group (*p* < 0.001) and patients without drug resistance (*p* = 0.049). According to Yang W. et al. [29], *HMGB1* is involved in the pathogenesis of TLE. The level of its expression in the hippocampal tissues is high. The studies of Walker L. E. et al. [34] and Kamaşak et al. [35] demonstrated that patients with refractory epilepsy with structural changes had a higher total level of *HMGB1* than the control group and the group with treatable epilepsy. The study by Kamaşak et al. [35] found a correlation between the severity of epileptic seizures and cytokines plasma level, including *HMGB1*, in children with drug-resistant epilepsy. These results are in contrast to our data, which is probably due to the heterogeneous sample of patients with different forms and durations of epilepsy.

A decrease in the level of pro-inflammatory cytokines, as observed in our study, was also recorded in cohort patients with TLE who had a longer course of the disease [36]. This can be explained by the fact that the long duration of the disease can induce an inadequate systemic anti-inflammatory immune response. Furthermore, a decrease in the level of pro-inflammatory cytokines is characteristic of patients taking sodium valproate and leviteracetam [31].

A decrease in the level of the pro-inflammatory cytokines *TNFa* and *HMGB1* may be associated with long-term chronic neuroinflammation in TLE, as well as with other pathophysiological mechanisms with a predominant involvement of T-cells; downregulation was observed in the peripheral blood of patients with mesial TLE [32] with a depletion of the immune defense mechanisms of the phagocytic link or endothelial factors [33,37].

*TNFa* is a pro-inflammatory cytokine [30] synthesized by neurons and glial or endothelial cells of the blood–brain barrier [36,38]; they possess neuromodulatory properties, thereby contributing to rapid changes in neuronal excitability [39]. *TNFa* affects seizure susceptibility in animal models [39,40]. Limbic seizures also cause an increase in *TNFa* expression in brain microglia and endothelial cells [41].

*BDNF* is a modulator of excitatory and inhibitory synaptic transmission [32]. A number of studies have demonstrated a dramatic increase in *BDNF* mRNA and protein expression in both animals and humans with epilepsy [37,42,43]. *BDNF* enhances the efficiency of excitatory synapses that connect the main neurons [43]. *BDNF*-mediated TrkB activation can also disrupt GABA-mediated inhibition [44]. Intrahippocampal infusion of *BDNF* or transgenic overexpression of *BDNF* or TrkB in transgenic mice resulted in an increased susceptibility to seizures or their severity [44]. Immunohistochemical evidence of increased TrkB activation, manifested as an increased pTrk immunoreactivity in the hippocampal mossy fiber pathway, has been demonstrated after seizure induction in rats and mice with various electrical stimulation models and various chemoconvulsants [45].

Decreased *BDNF* expression has also been observed in plasma in adult TLE patients compared with controls [42]. Presumably, the decrease in plasma BDNF levels in adult patients with TLE may be mediated by the use of AEDs. One study showed that phenobarbital, valproate, and phenytoin reduced the *BDNF* mRNA in the cingulate cortex, hippocampus, and thalamus [45]. A decrease in the level of *BDNF* may indicate the suppression of neuroplasticity and neurogenesis in TLE patients compared with healthy people.

Upregulation in cytokine expression is usually observed in the acute phase of epileptogenesis [46,47,48]. Despite the fact that in cases of chronic epilepsy, there is also an increase in the expression of cytokines [49,50], the duration is nevertheless not comparable in longitude with the disease duration in our study.

Neuronal death can directly lead to a decrease in cytokine synthesis and, consequently, the hypoexpression found in patients with TLE compared with controls. According to one of the hypotheses for the development of drug resistance in epilepsy [51], massive degeneration and remodeling of the neural network caused by seizures suppresses the endogenous anticonvulsant system and prevents the AED access to neuronal targets. Therefore, a decrease in the level of cytokines, caused by massive neuronal death and remodeling, can be a marker for the development of drug resistance.

A limitation of our study is that we were conditioned by heterogeneous plasma sample of patients with different forms and durations of epilepsy. Furthermore, the BBB disruption has not been calculated.

## 5. Conclusions

The concentration of the three markers of neuroinflammation in the blood plasma of patients with TLE was reduced compared with the control group.

Thus, *HMGB1* can be used as a biomarker of drug resistance in patients with TLE. In our study, a decrease in the concentration of *BDNF* and *TNFa* reflected the duration of TLE; particularly, the concentration of *TNFa* decreases with an increase in an onset duration of more than 10 years. The results of the study may indicate the acceptability of the use of *HMGB1* and *TNFa* as alternative markers of TLE, and the use of these markers may indicate the risk of developing drug-resistant TLE.

## Figures and Tables

**Table 1:** Clinical characteristics of the patients.

Characteristics	Drug-Resistant, n = 49	Drug Responsible, n = 117	P*
Sex	19 males, 30 females	50 males, 67 females	
Age at the time of observation, Me [LQ25; UQ75]	33.0 [28.0; 43.0]	31.0 [25.0; 41.0]	
Age of the onset, Me [Q25; Q75]	17.0 [8.5; 21.25]	20.0 [13.0; 31.0]	
Duration of the disease			
Less than 5 years n = 32	3	29	0.375
5–10 years n = 45	10	35	0.023
More than 10 years n = 89	36	53	0.4331
Seizure severity on the NHS-3 scale, Me [LQ; UQ], score	15.0 [14.0; 16.0]	12.0 [11.0; 15.0]	
TLE+HS	21	41	
TLE-HS	28	76	
Monotherapy	8	76	
Polytherapy	41	41	

P*—Mann–Whitney criterion; Me—median; LQ—lower quartile; UQ—upper quartile; n—amount of patients; NHS-3—National Hospital Seizure Severity Scale; TLE+HS—temporal lobe epilepsy with hippocampal sclerosis; TLE-HS—temporal lobe epilepsy without hippocampal sclerosis.

**Table 2:** The significant alteration in the concentrations of markers of neuroinflammation and neurodegeneration in the blood plasma of patients in the TLE and control groups.

Plasma Marker Concentration	TLE Patients (Drug-Resistant) Me [LQ; UQ]	TLE Patients (HS+) Me [LQ; UQ]	TLE Patients (Drug Responsible) Me [LQ; UQ]	TLE Patients, Me [LQ; UQ]	Control Group, Me [LQ; UQ]	P*
BDNF (ng/mL)	25.98 * [22.06; 31.29]	26.28 * [22.73; 31.27]	24.44 * [19.56; 32.62]	25.87 * [20.81; 32.17]	74.85 [45.11; 128.85]	<0.001
TNFa (pg/mL)	11.44 * [9.42; 16.45]	11.27 * [8.41; 18.68]	10.85 * [10.29; 18.35]	12.30 * [10.27; 20.95]	73.40 [56.42; 92.88]	<0.001
HMGB1 (pg/mL)	119.09 * [113.55; 126.93]	134.65 * [113.19; 156.11]	135.765 * [114.17; 159.53]	135.765 * [114.17; 159.53]	161.73 [136.34; 179.01]	0.034
NTRK-2 (pg/mL)	4.40 * [2.60; 5.50]	4.15 * [3.05; 4.65]	3.80 * [3.075; 4.83]	3.80 * [2.90; 4,70]	3.00 [2.40; 4.80]	0.365

*—Mann–Whitney criterion; Me—median; LQ—lower quartile; UQ—upper quartile; TLE—temporal lobe epilepsy; HS+—hippocampal sclerosis.

**Table 3:** The significant alteration in the concentration of the markers of neuroinflammation and neurodegeneration in the blood plasma of patients in the TLE and control groups, depending on the duration of the disease.

Plasma Marker Concentration	Duration Less Than 10 Years	Duration More Than 10 Years	Control Group	p1,2	p1,3 p2,3 p Common
*BDNF* (ng/mL)	26.50 [21.07; 32.87]	25.62 [19.83; 28.66]	74.85 [45.11; 128.85]	0.212	<0.001
*TNFa* (pg/mL)	14.30 [11.22; 26.85]	10.58 [8.70; 16.30]	73.40 [56.42; 92.88]	0.05	<0.001

**Table 4:** Area under the ROC curve with standard error and 95% confidence interval.

Marker	Area under the Curve	Standard Error	Asymptomatic Significance	95% Confidence Interval	
				Lower Limit	Upper Limit
*HMGB1*	0.664	0.073	0.033	0.520	0.807
*BDNF*	0.974	0.020	0.000	0.934	1.000
*TNFa*	0.894	0.040	0.000	0.816	0.972

## Data Availability

The data presented in this study are available on request from the corresponding author. The data are not publicly available due to internal regulations.
